# Case report: Canadian consensus on chlormethine gel use in mycosis fungoides-CTCL: literature review and real-world experience

**DOI:** 10.3389/fmed.2024.1474030

**Published:** 2024-12-16

**Authors:** Ivan V. Litvinov, Mohannad Abu-Hilal, Raed Alhusayen, Bernard Delisle, Jan Dutz, Sophie Guénin, Vincent Ho, Mark G. Kirchhof, Kevin Pehr, David Roberge

**Affiliations:** ^1^Division of Dermatology, McGill University, Montreal, QC, Canada; ^2^Division of Dermatology, McMaster University, Hamilton, ON, Canada; ^3^DermCare Clinic, Mississauga, ON, Canada; ^4^Department of Dermatology, CHU de Quebec, Laval University, Quebec, QC, Canada; ^5^Department of Dermatology and Skin Science, UBC and BC Children’s Hospital Research Institute, Vancouver, BC, Canada; ^6^Department of Dermatology, Mount Sinai Hopsital, New York, NY, United States; ^7^Department of Dermatology and Skin Science, University of British Columbia, Vancouver, BC, Canada; ^8^Division of Dermatology, Department of Medicine, The Ottawa Hospital, University of Ottawa, Ottawa, ON, Canada; ^9^Division of Dermatology, McGill University Jewish General Hospital, Lady Davis Intitute for Medical Research, Montreal, QC, Canada; ^10^Division of Radiation Oncology, University of Montreal, Montreal, QC, Canada

**Keywords:** chlormethine gel, mycosis fungoide, CTCL, skin directed therapy (SDT), Canada

## Abstract

Mycosis fungoides (MF) is the most common type of cutaneous T-cell lymphoma (CTCL), representing the majority of all lymphomas arising in the skin. The disease treatment focuses on managing symptoms and preventing disease evolution. To date, there is no gold standard for MF-CTCL treatment. Chlormethine, a DNA alkylating agent, is a long-known treatment for CTCL. The new chlormethine 0.02% gel (CL-gel) formulation provides proven efficacy and ease of application, improving patient compliance and outcome. The current consensus paper and real-world experience with CL-gel in the treatment of early-stage MF-CTCL may help meet the unmet need for treatments in Canada. A modified Delphi process comprised a virtual meeting and an online follow-up. A panel of 9 board-certified dermatologists with expertise in cutaneous lymphoma and 1 radiation oncologist discussed the systematic literature review results, drew from clinical experience and the opinion of the panel to adopt and agree on five consensus statements. The panel shared real-world patient cases to illustrate the use of chlormethine gel in a variety of patients across Canada. Five real-world patient cases were provided to illustrate the panels’ use of chlormethine gel.

## Introduction

Mycosis fungoides (MF) is the most common cutaneous T-cell lymphoma (CTCL), accounting for the majority of primary cutaneous lymphoma cases (1). While it is the most common form of CTCL, MF-CTCL remains a rare disease with only 2,510 cases documented from 1992 through 2010 in Canada (2). It typically affects older individuals with a median age at diagnosis of 55–60 years and a male-to-female ratio of 2:1 (1). MF-CTCL has an indolent, relapsing, and remitting clinical course with slow progression over years to decades (1). Early stages of the disease (IA-IIA) are characterized by red, scaly patches or plaques on the skin (1). These scaly patches and plaques may resemble other dermatological conditions such as eczema or psoriasis; however, unlike these other conditions, MF-CTCL has a predilection for the buttocks and other sun-protected areas (1). As the disease progresses to more advanced stages (IIB-IVB), patches may evolve into infiltrated and ulcerated plaques or tumors that infiltrate the blood (1). Further progression of MF-CTCL may also lead to lymph nodes and visceral organ involvement (1).

Diagnosis of MF-CTCL is made based on clinical exam findings, multiple skin biopsies, immunostainings, and molecular studies (1). As MF-CTCL resembles a variety of other skin conditions, diagnosis of the disease is often delayed for years (1). In advanced stages of the disease, peripheral blood flow cytometry, TCR gene rearrangement studies and imaging are necessary for staging. The prognosis of MF-CTCL largely depends on the clinical stage at diagnosis, extent of cutaneous involvement and the presence of extracutaneous disease. Early-stage (IA-IIA) MF-CTCL has little impact on life expectancy while advanced disease (IIB-IVB) has a 5-year survival rate of only 52% (3). From 1992 to 2010, the number of deaths attributed to MF-CTCL in Canada, was approximately 0.4 deaths per million (2). Treatment of MF-CTCL is largely directed at preventing the evolution of the disease, symptom control, and improvement of quality of life (1). Early-stage MF-CTCL is treated with skin-directed therapies (SDT), which include topical treatments such as topical corticosteroids (TSC), retinoids, imiquimod, chlormethine, carmustine, phototherapy, narrow band UVB, and ultraviolet A (PUVA) among other topical agents addressed further in the text (4). Advanced MF-CTCL is treated with systemic therapies, often in combination with SDTs (1). To date, MF-CTCL is considered a largely incurable disease and follows a relapsing and remitting course, often refractory pattern. The only potential definite cure is stem cell transplantation for suitable candidates (1).

Chlormethine (CL) is a synthetic bifunctional DNA-alkylating agent that inhibits rapidly proliferating T-cells in the skin (5). *In vitro*, CL induces significant double-stranded DNA breaks in MF-CTCL clonal malignant skin T-cells, while sparing bystander skin or blood T cells (5). In addition, malignant T cells have impaired DNA repair mechanisms with suppression of homologous recombination repair genes (FANC1, FEN1, and BRCA2) with increased CASP3, which makes them more susceptible to apoptosis (5). The DNA-alkylating action of CL may also confer an immunostimulatory effect in the tumor microenvironment which upregulates cytotoxic T cell actions to further support CL anti-tumor effects (6). CL exists in aqueous solutions, compounded ointments, or gel formulations. The aqueous and ointment preparation of CL have been used for decades; however, studies have shown that CL gel has a faster time to response likely due to its increased release rate making the drug delivery more efficient with higher response rates (7). The gel penetrates the epidermis within 2–4 h and the dermis within 4–6 h, with no evidence of systemic absorption (7).

In Study 201, a pivotal, randomized observer-blinded controlled clinical trial, MF-CTCL Composite Assessment of Index Lesion Severity (CAILS) response rates for CL gel versus ointment were 58.5 and 47.7%, respectively (8). This trial demonstrated that CL 0.02% gel was numerically more effective in reducing number and severity of lesions than the CL ointment preparation (8). The results of study 201 led to CL gel federal drug administration (FDA) approval in the United States in 2013 for adult patients with stage IA-IB MF-CTCL who have received prior SDT (9). Following this, major international guidelines such as the British Association of Dermatologists/UK Cutaneous Lymphoma Group, European Society of Medical Oncology (ESMO), and European Organisation for Research and Treatment of Cancer (EORTC), recommended topical CL as the first-line agent for MF-CTCL stages IA, IB, and IIA (10). The SDT has a predictable response and favorable side effect profile that allows for long-term use and prolonged efficacy after discontinuation (9, 10). CL gel was approved by Health Canada in 2021 as a topical antineoplastic agent for MF-CTCL stages IA and IB (11).

Common side effects of CL gel include dermatitis, pruritus, skin infections, ulceration, or cutaneous hypersensitivity reactions (11). In clinical trials, discontinuation due to adverse events occurred in 22% of CL gel treated patients and 18% of patients with the comparator (11). CL gel had a higher rate of discontinuation due to skin irritation which can be treated with topical corticosteroids (11, 12).

While guidelines exist for the treatment of MF-CTCL, there are few randomized clinical trials investigating treatments for this rare disease. CL gel is a topical antineoplastic agent with phase II clinical trial and real-world data, indicated for patients in Canada with stage IA-IB MF-CTCL who have received prior SDT.

The current consensus paper and real-world experience with CL-gel in the treatment of Canadian patients suffering from MF-CTCL is to provide insights into its role as topical monotherapy in the early-stage disease or adjunctive therapy to systemic treatment in more advanced stages of MF-CTCL.

## Methods

### Systematic literature search

To address the question of what do we know from the literature about MF-CTCL topical treatment with CL-gel, literature searches were performed by a medical researcher using PubMed and Google Scholar (secondary source), first evaluating the title, the abstract, and then the full article. Search terms are detailed in Table 1. The searches included English language articles (randomized controlled trials, other clinical studies, guidelines, consensus articles, and review articles) published between January 2010 and July 20th, 2023. The searches yielded 62 articles comprising 27 clinical studies and 35 reviews. The clinical studies were graded using a grading method detailed in Begolka et al. (13)

**Table 1:** Search terms.

Search terms	Group 1	Group 2
	“Mycosis fungoides”[MeSH Terms] OR “Mycosis Fungoides” [Text Word] AND “chlormethine gel” [Text Word] OR “chlormethine” [Text Word] OR “mechlorethamine” [Text Word] OR “topical monotherapy with chlormethine gel” [tiab:~0] OR “concomitant therapy with chlormethine gel” [tiab:~0] OR “treatment regimen” OR “skin-related side effects”	“Mycosis fungoides, chlormethine, mechlorethamine”

A panel of 9 board-certified dermatologists with expertise in cutaneous lymphoma and 1 radiation oncologist from four different provinces (Quebec, British Columbia, Alberta, Ontario) used a modified Delphi process comprising a virtual meeting and an online follow-up. The process entailed preparing the project, selecting the panel, and using the systematic literature search results to inform 11 draft statements.

During a virtual meeting on September 14th, 2023, the panel discussed the systematic literature review results and evaluated the draft statements in a workshop. In a plenary discussion, drawing from clinical experience and opinions, the panel adopted five statements to provide clinical guidance on CL-gel as topical monotherapy in early stage disease or adjunctive to systemic treatment for Canadian healthcare providers treating patients suffering from MF-CTCL. The panel then voted, and all five statements (Table 2) met the criteria for consensus (>80%). After preparing the manuscript, the individual advisors reviewed it and agreed with its content and publication.

**Table 2:** Consensus statements for treatment of mycoides fungoides with chlormethine gel.

Statement no.	Statement
1	Currently, there are limited topical options in Canada for early-stage MF-CTCL. Chlormethine gel (CL gel) is a Health Canada-approved topical alkylating agent that inhibits malignant T-cells involved in MF-CTCL. A phase II clinical trial and real-world data demonstrate safety and efficacy as a treatment option for adults with MF-CTCL.
2	CL gel is the skin-directed therapy with the highest level of recommendation for early-stage (Stages IA, IB, and IIA) MF-CTCL in the European Society of Medical Oncology guidelines, the European Organisation for Research and Treatment of Cancer, and others.
3	CL gel’s clinical activity is restricted to the skin, making it particularly effective in early-stage disease, though it can also be used as adjunctive therapy for advanced disease.
4	CL gel used for MF-CTCL has a predictable and durable response with prolonged efficacy. It provides a beneficial option for treating patients with MF-CTCL in Canada.
5	Skin-related side effects of CL gel, such as dermatitis, can generally be managed through appropriate strategies.

### Real-world patient cases

Four expert panelists volunteered to share real-world patient cases involving their use of CL gel to treat MF-CTCL. Two cases were offered by each expert. During the consensus meeting, the panel shared real-world patient cases to illustrate the use of CL-gel in a variety of patients across Canada. The presented cases discussed patient presentation, clinical challenges, and treatment outcomes. These cases served as a forum for dermatologists to exchange ideas and reflect on how to provide the best care for MF-CTCL patients.

The real-world cases (RWC) characterize dermatologists’ real-world experience (i.e., how are experienced specialists in practice using the product and how their patients are doing on the regimen) when using the treatment. The RWC did not require ethics committee approval as it reports real-world experience and does not make statements on the efficacy or safety of the treatment. The RWC was conducted complying with good clinical practice (GCP). All authors obtained written informed consent from the individuals who participated in the RWC. The participants in the RWC series allowed the recording of their photographs to be used for the manuscript and its publication.

## Results

*Statement 1*: Currently, there are limited topical options in Canada for early-stage MF-CTCL. CL gel is a Health Canada-approved topical alkylating agent that inhibits malignant T-cells involved in MF-CTCL. A phase II clinical trial and real-world data demonstrate safety and efficacy as a treatment option for adults with MF-CTCL.

To date, there are limited options for MF-CTCL treatment in Canada. Thus, treatment regimens are largely based on consensus guidelines such as those put forth by the EORTC (14). The EORTC divides MF-CTCL treatments into three categories: Expectant policy (watch and wait), Skin-Directed Therapies (SDT), and Systemic Therapies (14).

### Expectant policy

For patients with low-risk stage IA MF-CTCL, an expectant or watch-and-wait policy may be sufficient to manage these patients (14). It is estimated that there is only a 10% risk of progression over 10 years in stage IA MF-CTCL (14). In PROCLIPI study, first-line treatments were analyzed in patients with newly diagnosed MF-CTCL (stages IA-IIA) (15). In this 395 patient cohort, expectant observation was only used in 7.3% of cases and 45% of patients treated initially with expectant policy received subsequent treatment after a median of 7.5 months (range 3–34 months), indicating an eventual need for treatment (15). Thus, while expectant policy may be used for short while, the general consensus is that most patients (81.5%) are started on SDT for first-line therapy (15).

Appropriate patient counseling and careful monitoring is required to determine a treatment plan for low-risk, early-stage MF-CTCL. While expectant policy may be considered upon initial diagnosis, SDT will prevent progression and ease patient discomfort.

### Skin-directed therapies

SDTs are localized cutaneous treatments recommended as first-line agents for MF-CTCL Stages IA, IB, and IIA. TCS are the most commonly prescribed SDT (14). Despite little evidence for use of TCS in MF-CTCL, TCS are considered an economical and safe option to alleviate symptoms of itch/burning for early-stage MF-CTCL (14). However, TCS are palliative and not curative and can lead to long-term adverse effects (14). Originally derived from nitrogen mustard gas, topical CL is an alkylating agent that has long been used as a topical treatment for MF-CTCL with variable efficacy in various vehicles (14). Topical bexarotene is a retinoid that is available in a 1% gel formulation approved for MF-CTCL stages IA-IB (14). In two prospective trials evaluating bexarotene, response rates varied between 44 and 63% at the study end-point (14). Ultraviolet phototherapy such as PUVA, localized radiotherapy, and total skin electron beam therapy (TSEB) are also treatment options; albeit associated with significant long-term toxicity with prolonged use (14).

### Systemic therapies

Systemic therapies are generally recommended as first-line agents for MF-CTCL stage IIB and second-line agents for MF-CTCL stages IA, IB, and IIA (14). Common systemic therapies include methotrexate, retinoids, interferon-*α* (IFNα), targeting immunotherapy, extracorporeal photochemotherapy, select forms of chemotherapy, hematopoietic stem cell transplantation, and histone deacetylase inhibitors (14). These agents may be used in monotherapy or in combination with other agents and SDTs.

There are no gold standard treatments for MF-CTCL and limited SDT options. Even for the widely prescribed TCS, only one uncontrolled study by Zackheim et al. demonstrates that high-potency TCS, clobetasol propionate, was effective in 79 stage IA/B MF-CTCL patients (16). Further, topical bexarotene has two clinical trials, which showed variable efficacy (14). In addition, bexarotene is highly teratogenic, which restricts its use in certain populations (14).

Topical CL is an alkylating agent with many large, uncontrolled studies demonstrating its efficacy in various compounded formulations. Early CL preparations were aqueous or compounded ointment-based formulations, which were difficult to apply and less efficacious than the newer gel formulation (14). Compounded ointments were also available in some pharmacies and found to have limited stability (14). Alternatively, in addition to being easy to apply and fast-drying, the CL gel demonstrated non-inferiority (58.5%) to the chlormethine ointment (47.7%) in a pivotal phase II study (Study 201) (8). Study 201 demonstrated higher response rates for CL 0.02% gel compared to CL ointment at primary endpoint of CAILS in a 260-patient randomized, clinical trial (8). The response rate also increased with time, with 76% of patients achieving a complete response by week 52 compared to 46% in week 24 (8).

Adverse events were experienced by 61.7 and 50.4% of patients who received the gel and ointment, respectively (8). The most common adverse event was dermatitis, experienced by more than half of the patients (8). Dermatitis was managed by suspension of reduction of CL treatment and use of emollients or oral antihistamines (8). Treatment was resumed upon improvement at a reduced frequency (once every 3 days) (8).

Following the results of Study 201, CL (0.02%) gel was approved by Health Canada for treatment of stage IA and IB MF-CTCL in adult patients who have received prior SDT. Since its approval, the CL gel has been evaluated in a multitude of small and large observational and case series studies (17–19). Notably, the PROVe Study demonstrated that in 298 patients, under real-world conditions without any specific visit schedules or clinical assessments, CL gel was an efficacious and important therapeutic option for MF-CTCL patients (20).

*Statement 2*: CL gel is the skin-directed therapy with the highest level of recommendation for early-stage (Stages IA, IB, and IIA) MF-CTCL in the European Society of Medical Oncology (ESMO) guidelines, the European Organisation for Research and Treatment of Cancer (EORTC), National Comprehensive Cancer Network, and others.

The 2018 World Health Organisation- EORTC classifies CTCL as primary cutaneous lymphomas that exist as MF-CTCL, MF-CTCL variants, or Sezary syndrome (SS) (21). SS is closely related to MF-CTCL and shares clinical and histopathological similarities (21). However, while MF-CTCL is largely indolent, SS is characterized by aggressive clinical behavior presenting as a triad of generalized erythroderma, lymphadenopathy, and other systemic manifestations (21). In rare cases, MF-CTCL may evolve to SS for which there is only a 36% 5-year disease-specific survival rate (22). MF-CTCL and SS are classified using a modified TNMB-classification system in which disease stage is based on skin, nodal, and visceral organ or blood involvement (Table 3) (14, 21, 22). From these stages (IA-IVB), a 5-year survival prognosis is predicted (22). Early stages IA and IIA have high 5-year survival rates, 98 and 89%, respectively (22). Thus, treatment at early stages is aimed at preventing evolution and progression of disease to SS and improving symptoms and quality of life.

**Table 3:** Clinical stages of mycosis fungoides and sézary syndrome with disease-specific 5-year survival rate and treatment recommendations.

Stage	T	N	M	B	Prognosis: 5-year survival rate (%)	ESMO/EORTC first-line treatment recommendations
IA	1	0	0	0 or 1	98	Expectant policy, TCS, topical chloramine, UVB, PUVA, localized RT
IB	2	0	0	0 or 1	89	
IIA	1 or 2	1 or 2	0	0 or 1	89	
IIB	3	0–2	0	0 or 1	56	Systemic therapies: retinoids, IFNα, TSEB, low dose MTX, localized RT, mono-chemotherapy Systemic therapies should be combined with SDT (ESMO)
IIIA	4	0–2	0	0	54	Same as IIB with addition of extracorporeal photopheresis
IIIB	4	0–2	0	1	48	
IVA1	1–4	0–2	0	2	41	Chemotherapy, allogeneic stem cell transplantation
IVA2	1–4	3	0	0 or 2	23	
IVB	1–4	0–3	1	0 or 2	18	

Adapted from Kim et al. (20).

Treatment recommendations are stratified by disease stage in the EORTC MF-CTCL classification system (Table 3) (14, 21). EORTC first-line recommendations for MF-CTCL Stages IA, IB, and IIA are expectant policy, TCS, UVB, PUVA, localized radiotherapy, and topical CL (14, 21). The highest level of evidence exists for CL, UVB, and PUVA. Similarly, ESMO also recommends expectant policy, TCS, UVB, PUVA, local radiotherapy, and topical CL for first-line treatment of stage IA, IB, and IIA MF-CTCL (Table 3) (23). Unlike EORTC, ESMO consensus indicates that topical CL has the highest level of evidence for use (23). It must also be noted that TCS provide an effective and cost-effective first-line approach. However, few randomized clinical trials have been conducted on TCS in MF-CTCL, TCS have long been used in clinical practice with effective results (16). However, TCS is not appropriate for long-term use as it may lead to straie, skin atrophy, hypopigmentation, skin irirration, and skin infections. ESMO also indicates that SDT should be combined with systemic therapies such as retinoids or IFNα in stage IIB MF-CTCL (23).

*Statement 3*: CL gel’s clinical activity is restricted to the skin, making it particularly effective in early-stage disease, though it can also be used as adjunctive therapy for advanced disease.

Topical application of CL undergoes rapid metabolization via hydrolysis and demethylation within minutes of contact with the body’s hydrophilic surface (24). *In vitro* release testing reveals that rate of CL release from the gel formulation is significantly greater than the ointment-based formulation (24). Both the rate and cumulative amount of CL released were significantly greater from the CL gel than from the ointment (24). CL gel was also shown to exert its primary activity at the epidermal layer (24). It has a mean flux of 10.8 ng/cm^2^/h in the epidermal membrane with its peak occurring at 2 h post-application (24). Penetration testing showed that mean residual CL on the surface of skin after 24 h was 21 ng (1.3% of applied dose) in epidermis and undetectable in the dermis (24). This indicates that the CL-gel action is restricted to the epidermis, which explains its effectiveness in early-stage MF-CTCL solely localized to the epidermis (24).

To ensure safety of CL gel, bioanalytic assays were performed from patient samples from study 201 to determine CL concentrations in the plasma. The assays found that there was no measurable evidence of CL in plasma samples after topical once-daily application of 0.02% CL gel or ointment, or 0.04% CL gel. Laboratory monitoring of hematologic parameters also showed no abnormalities or changes over time. In summary, CL uniquely targets the epidermis and dermis following topical administration, with no evidence of systemic absorption.

The lack of systemic absorption with CL gel positions it as a valuable adjunctive treatment for systemic therapies due to minimal risk of drug–drug interactions and systemic toxicity. Both ESMO and EORTC recommend combining SDTs such as topical CL-gel with systemic therapies in stage IIB-III MF-CTCL (14, 21–23). The literature also confirms that CL-gel is frequently and successfully used in combination with other SDT and systemic therapies (25). In the real-world PROVe study, combination treatment regimens were common, with 78% of patients using CL-gel combined with other SDT and 30% of patients using systemic therapies combined with CL gel (20). TCS, phototherapy, and oral bexarotene were the most commonly combined therapies with topical CL gel. Another real-world clinical experience examined 23 patients’ responses to CL-gel (26). In this study, 11 of the 12 patients received CL gel in combination with either methotrexate (MTX) or pegylated IFNα-2A (26). In 5 patients with stage IIB MF-CTCL, CL gel was added to treat localized tumor lesions and saw a significant clinical response (26). Case studies have also shown the successful use of CL-gel combined with phototherapy, retinoids, IFNα, acitretin, and mogamulizumab (27). Lastly, CL-gel can be an adjunctive maintenance therapy for TSEB and has been shown to prolong the time needed for upstaging by approximately 22 months (28).

*Statement 4*: CL gel used for MF-CTCL has a predictable and durable response with prolonged efficacy. It provides a beneficial option for treating patients with MF-CTCL in Canada.

Although there is a potential risk for dermatitis, CL gel remains a safe and efficient treatment that may be used long-term as maintenance (1–4 times weekly) or active treatment (once daily). In Study 201, the duration of response was maintained through month 12 in 86% of patients in the gel arm and 82% in the ointment arm (8). It was estimated that at least 90% of the responses would be maintained for at least 10 months after the study endpoint (8). Durable responses with active use of chlormethine gel (once daily) make the gel a reliable treatment option (8). However, few studies have documented the continued efficacy after gel discontinuation.

A Stanford group reported an average 12-month time period until relapse in 203 MF-CTCL patient-responders who discontinued topical CL treatment (aqueous solution) (29). The group also noted that patients who used a maintenance regimen had longer-lasting responses during maintenance therapy than those who did not continue a CL maintenance therapy regimen (29).

Case reports and expert experience demonstrate that reducing the frequency of CL gel application as maintenance treatment can delay the progression of disease and reduce the frequence of side effects such as skin irritation (28, 29). CL gel is an optimal maintenance therapy as it is safe to use long-term, accessible, and easy to apply. In one study, reducing CL gel application to 1–4 times weekly maintained approximately half of patients in a stable disease state (30). In a case series, two patients had maintained response 1–5 months after stopping CL gel application (31). While no large-scale studies exist to investigate the efficacy of CL gel after discontinuation, clinical experience suggests that CL gel is a feasible and reliable maintenance treatment for patients who may titrate up or down the dosage to maintain initial response (32).

*Statement 5*: Skin-related side effects of CL gel, such as dermatitis, can generally be managed through appropriate strategies.

Despite high efficacy rates, CL use is accompanied by a high discontinuation rate due to adverse effects such as skin irritation. With both the gel and ointment preparations, CL application leads to contact dermatitis characterized by skin irritation, erythema, and pruritus in 50–60% of patients (8). Mild-to-moderate dermatitis may not require suspension of treatment and can be palliated with TCS or decreased dosing frequency (3 times per week) (9, 11). Severe dermatitis requires discontinuation until improvement is seen (11). Dermatitis reactions vary from delayed hypersensitivity reactions to non-delayed hypersensitivity reactions such as irritant contact dermatitis, bullous reactions, and burning. Improving differentiating and understanding the various reactions will aid in patient compliance and outcomes. Hypersensitivity to CL or any ingredient in the gel is a contraindication for treatment (11).

To address the high discontinuation rates, some experts have recommended decreasing the application frequencies or titrating up the frequency of use. However, decreased CL use will inevitably lead to slower treatment responses and decreased efficacies. In addition, the efficacy of CL gel increases over time with continual use with some peak responses occurring between 8 and 10 months; thus, tolerance and adherence to treatment is crucial for positive outcomes (8, 33). Other appropriate treatment strategies for CL gel include choice of application site, proper patient selection, and adjunctive therapies to mitigate adverse events. Dermatitis can occur at any skin site; however, the risk for dermatitis is increased if applied to the face, genital area, anus, or skin folds (11). Any site may be affected by dermatitis. In general, application to mucous membranes should be avoided. Thus, choosing skin sites at lower risk of dermatitis may improve patient outcomes.

The prospective, randomized, controlled MIDAS study investigated the ability of topical triamcinolone ointment to prevent dermatitis associated with CL gel (12). Twenty-five patients with early-stage MF-CTCL were followed for 12 months (12). Two similar MF-CTCL lesions were chosen on each patient (11). One lesion was treated with once-daily CL gel for 4 months while the second lesion was treated with both CL gel and triamcinolone 0.1% ointment, daily (12). The study identified that the peak of adverse reactions occurred at months 2 and 3 and those lesions treated with the TCS had decreased contact dermatitis and greater improvement in quality of life (12). Importantly, the coadministration of triamcinolone 0.1% and CL gel did not affect the efficacy of the therapy (12). Further analysis of the MIDAS study also revealed that patients who developed allergic contact dermatitis to CL-gel may have an allergic-type phenotype that triggers reactions to common allergens unrelated to the CL-gel (34). Improving patch testing sensitivity, patient education, and understanding of the dermatitis reaction is necessary to improve the tolerability and clinical utility of CL-gel.

### Real world cases

CL gel is a first-line, effective SDT for early-stage MF-CTCL in Europe and the United States. Recently, the SDT was also approved in Canada. The real-world cases presented here demonstrate how the gel was used for various patients with MF-CTCL across Canada. The consensus advisors reported on real-world patient cases that illustrate the clinical experiences that have informed their expert consensus statements. The cases covered diverse patient presentations, clinical challenges, and treatment outcomes, which were discussed during the virtual meeting.

#### Case 1. CL gel use in multi-modality MF-CTCL therapy

**Figure 1:**
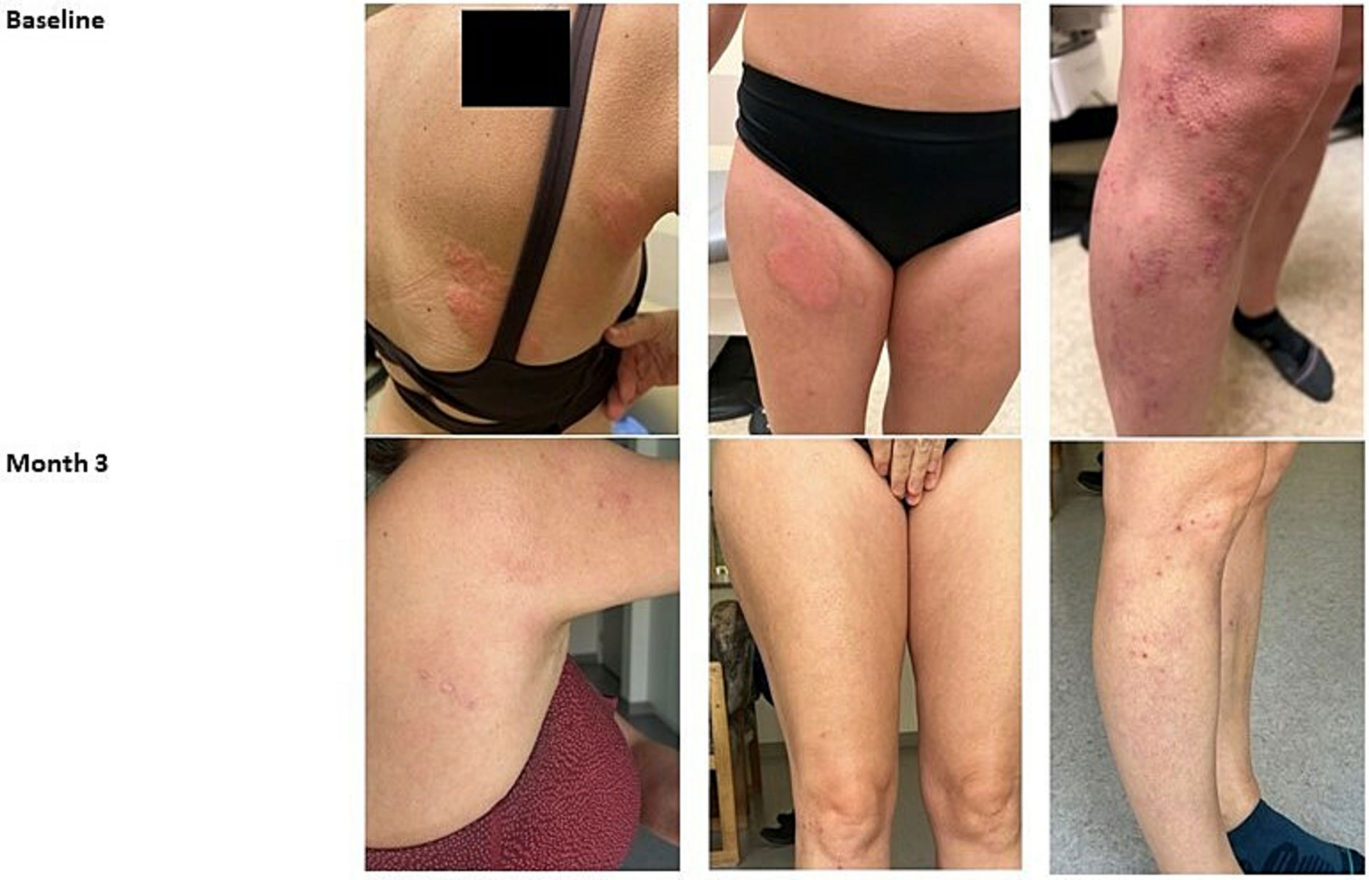
Case 1_48-year-old female. Forty eight-year-old woman with folliculotropic MF-CTCL before and after 3 months treatment with CL gel, UVB, and acitretin multi-modal therapy (Photographs courtesy of Jan Dutz MD).

A 48-year-old woman presents with an 8-year history of folliculotropic MF-CTCL, a variant of classic MF, and concurrent large granular lymphocytic lymphoma. Prior to her presentation, she had received a variety of diagnoses such as facial rosacea, tumid lupus, and atopic dermatitis for which she had tried and failed a variety of treatments such as prednisone, dapsone, isotretinoin, bilastine, ivermectin cream, antibiotics, TCS, and metronidazole cream. Upon work-up and examination, she was found to have 15% body surface area (BSA) MF and positive clonality analysis in skin and blood. She was started on narrow-band UVB phototherapy (3 times per week), acitretin 10 mg daily, CL gel, and gabapentin as needed for the itch. The patient was instructed to apply CL gel only to lesioned skin and to avoid applying the gel to the face. The patient saw an excellent response to lesions on the trunk and limbs where CL gel was applied. Within 3 months, the patient saw near resolution of the erythematous patches and plaques covering her back and lower extremities (Figure 1). This case demonstrates successful use of CL gel in a multi-modal MF-CTCL therapy approach in a patient who had been previously refractory to multiple previous treatments. The case also demonstrates appropriate patient counseling on where to apply CL gel to avoid sensitive areas.

#### Case 2. Adjusted CL gel dosing for severe contact dermatitis

**Figure 2:**
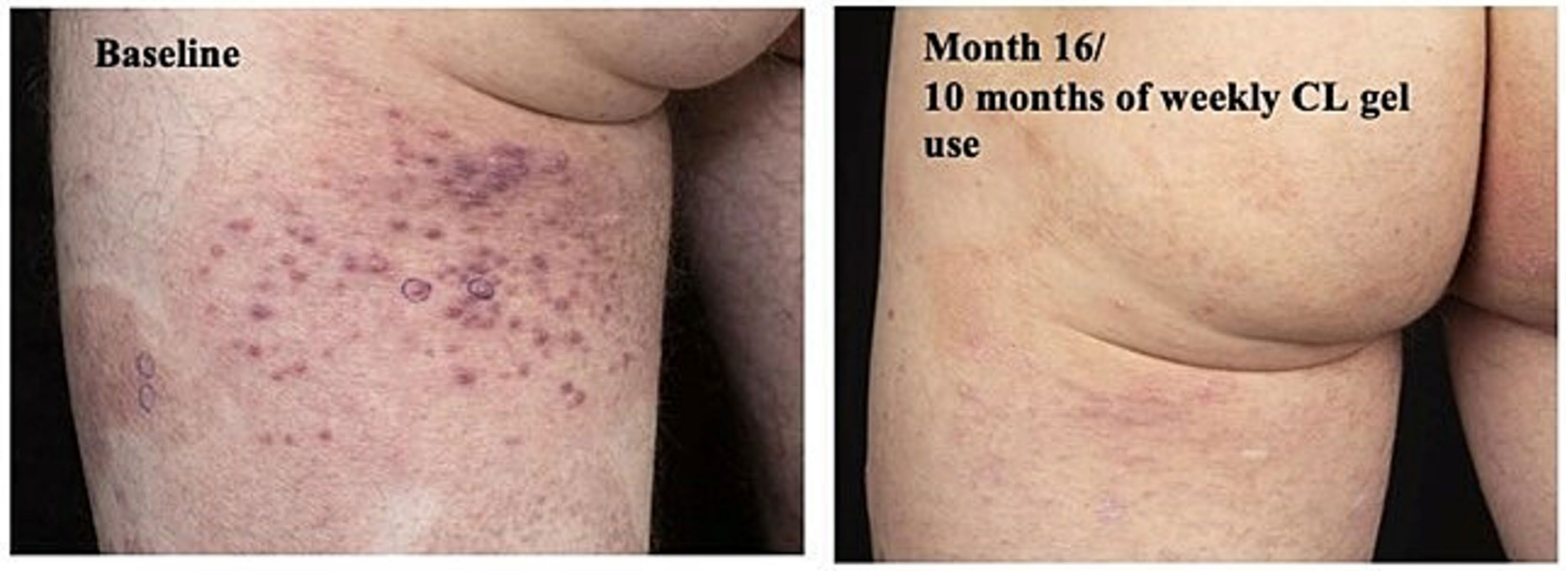
Case 2_43-year-old man. Forty three-year-old man with MF-CTCL on posterior thighs before and after 10 months of adjusted weekly CL gel use (Photographs courtesy of Bernard Delisle MD).

The 43-year-old male with a history of childhood-onset atopic dermatitis, presented with progressive pruritic plaques covering 20% of his body. The plaques started on his wrist and slowly evolved to involve his arms, legs, buttocks, palms, and soles. Prior to presentation, the patient had seen outside providers who had trialed him on TCS, phototherapy, cyclosporine, methotrexate, tacrolimus, dupilumab, acitretin, apremilast, and risankizumab. The patient failed to respond to any of these therapies. Clinical presentation and correlated biopsy led to a diagnosis of MF-CTCL Stage IB. At this time, CL gel had not yet been approved for use in Canada. Thus, through the special access program, hospital drug committee, and procurement of a letter of authorization from Health Canada, the patient was able to obtain CL gel for once-daily use. Unfortunately, after 6 weeks of use, the patient developed a severe contact dermatitis. Thus, CL-gel was stopped and the patient was prescribed topical clobetasol ointment and antihistamines for 1 week. Once the patient began to see improvement, he was restarted on once weekly CL-gel application. After 10 months on weekly CL gel, the patient saw remarkable improvement in his MF-CTCL lesions (Figure 2).

This case suggests that patients who experienced contact dermatitis may still achieve an adequate skin response. Appropriately evaluating and managing contact dermatitis after CL gel treatment is essential to maximize the chance for patients to remain on the efficacious treatment.

#### Case 3. Use of CL gel in combination with systemic therapies for stage IB MF-CTCL

**Figure 3:**
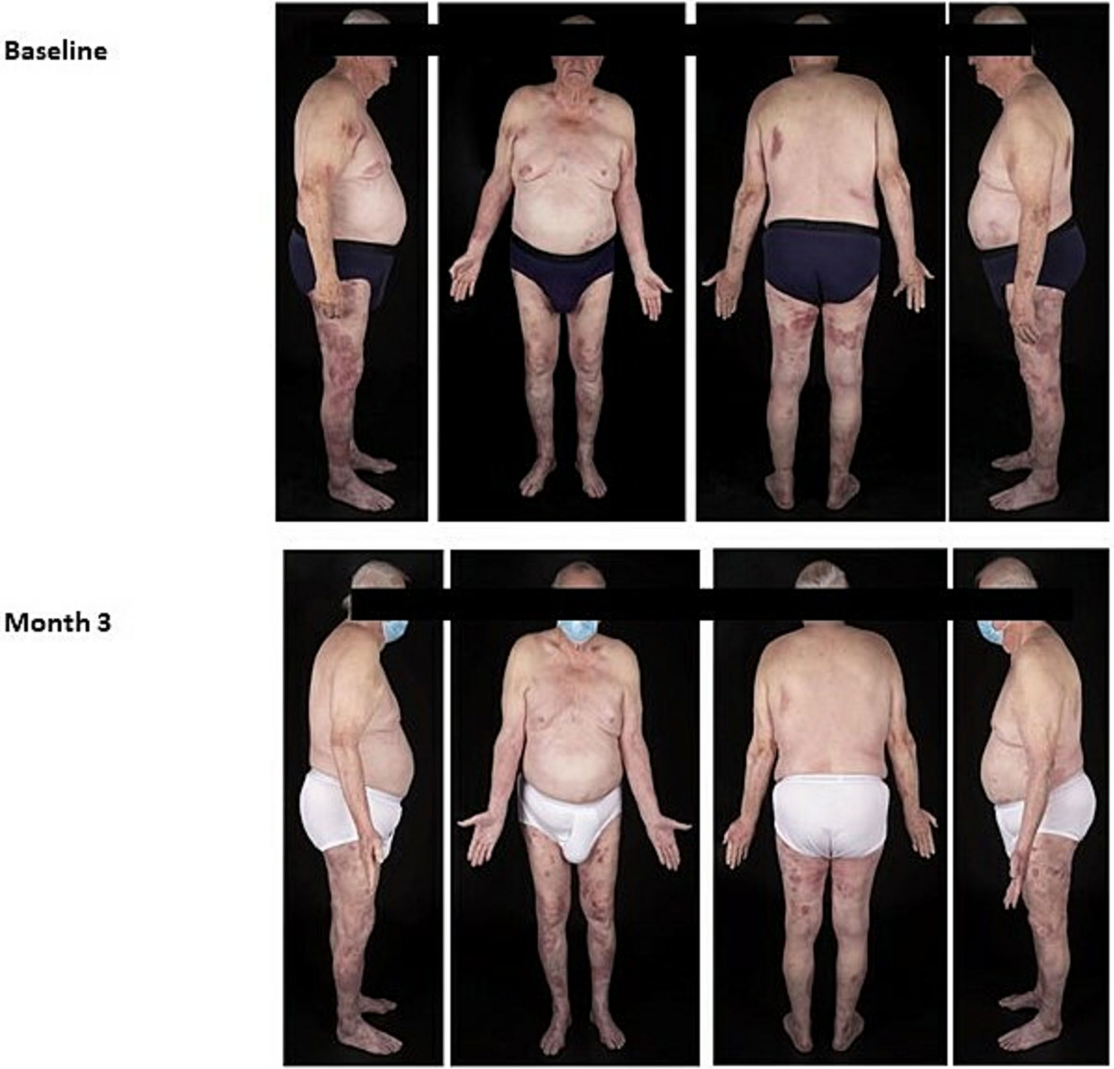
Case 3_84-year-old man. Eighty four-year-old male before and after 6-months of daily CL gel use in combination with methotrexate (photographs courtesy of Bernard Delisle MD).

An 84-year-old man with a history monoclonal gammopathy of uncertain significance (MGUS) and osteoporosis presented with a 3-year history of MF. The patient was initially diagnosed with Stage IB MF-CTCL and saw approximately 80% clinical improvement over 2 years with narrow-band UVB phototherapy. However, after 2 years, the patient failed to maintain his initial response. Thus, at this time, the patient was started on compounded NM in Aquaphor. The patient did not tolerate the compounded NM due to it being “too messy.” At this time, the patient was switched to a TCS cream with mixed results and potential risk of worsening osteoporosis. After a few months, the patient was proposed to try MTX and CL gel combination treatment as he had not been responding to topical monotherapy. Despite having some gastrointestinal side effects from the MTX, the patient tolerated the combination treatment well and saw significant improvement in his condition (Figure 3). The CL gel treatment was successful for a period of 34 months and the patient reported that the gel was more convenient and pleasant than the compounded formulation. Unfortunately, after 34 months, the disease evolved from a cutaneous stage to leukemic phase and started on another systemic therapy.

#### Case 4. Use of TCS and CL gel as active and maintenance MF-CTCL therapy

**Figure 4:**
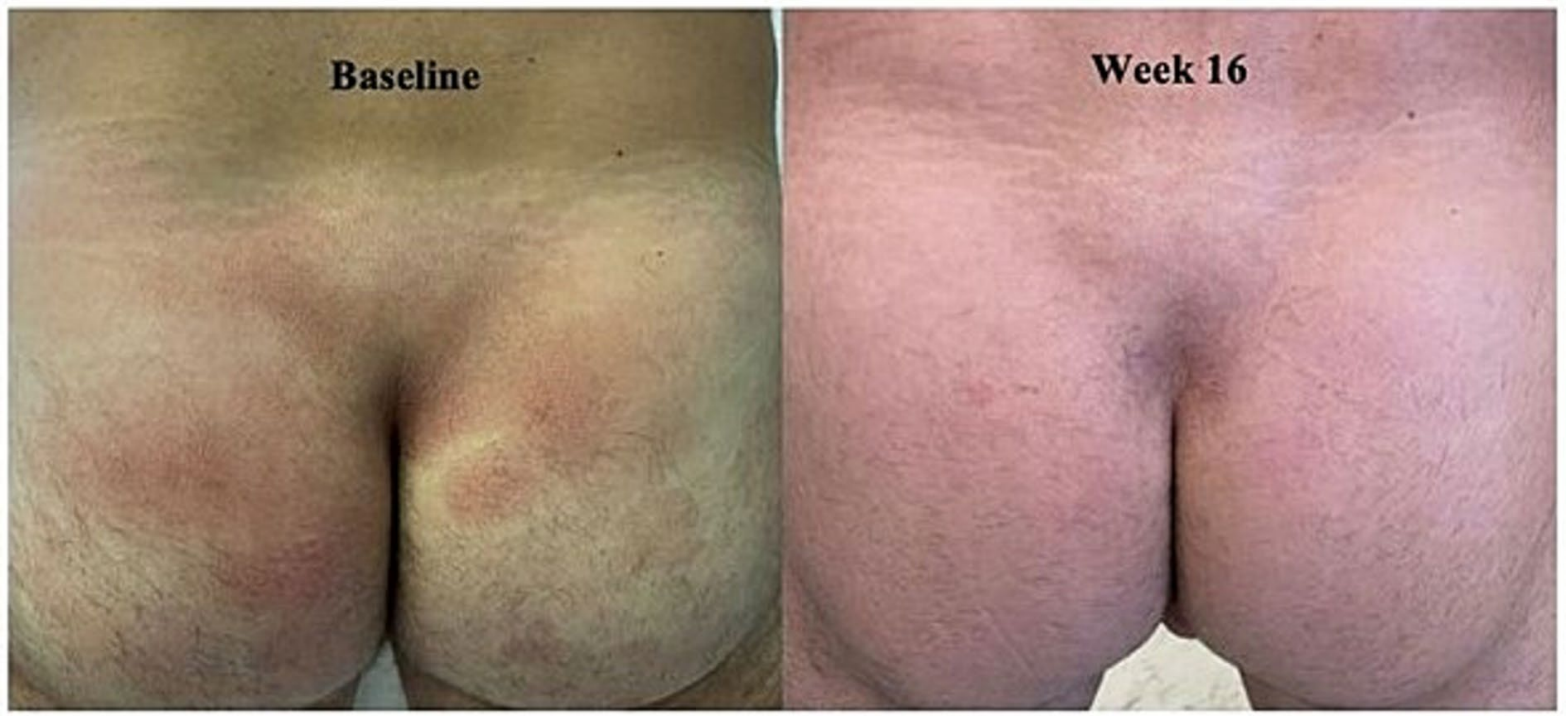
Case 4_46-year-old man. Forty six-year male with Stage IB MF-CTCL before and after 16 weeks on TCS and CL Gel treatment regimen (photographs courtesy of Mohannad Abu-Hilal).

The 46-year-old male with a history of stage 2A melanoma and a squamous cell carcinoma on his nose, presented with newly diagnosed MF-CTCL stage IB on the buttocks, lower trunk and thighs. The patient had these lesions for 5 years prior to diagnosis and had failed TCS, mometasone prior to presentation. Upon presentation, the patient had patches and “thin” plaques on his lower back, buttocks, and thighs with approximately 15% BSA. His biopsy showed marked epidermotropism of atypical, hyper-convoluted lymphocytes without spongiosis. Immunohistochemistry confirmed a T-cell (CD3) infiltrate with a CD4 to CD8 ratio of 6:1 and loss of CD7. Given the patient’s busy work schedule, the patient was not a candidate for UVB phototherapy. Instead, the patient opted to follow a daily CL gel and clobetasol ointment (every other day) regimen. The patient saw significant improvement after 16 weeks on this regimen (Figure 4). The patient did not experience any adverse effects on this regimen and remained fully in remission at week 24, his last visit. The patient continues the clobetasol/CL gel routine as needed as maintenance therapy.

#### Case 5. Use of CL gel in a medically-complex patient

**Figure 5:**
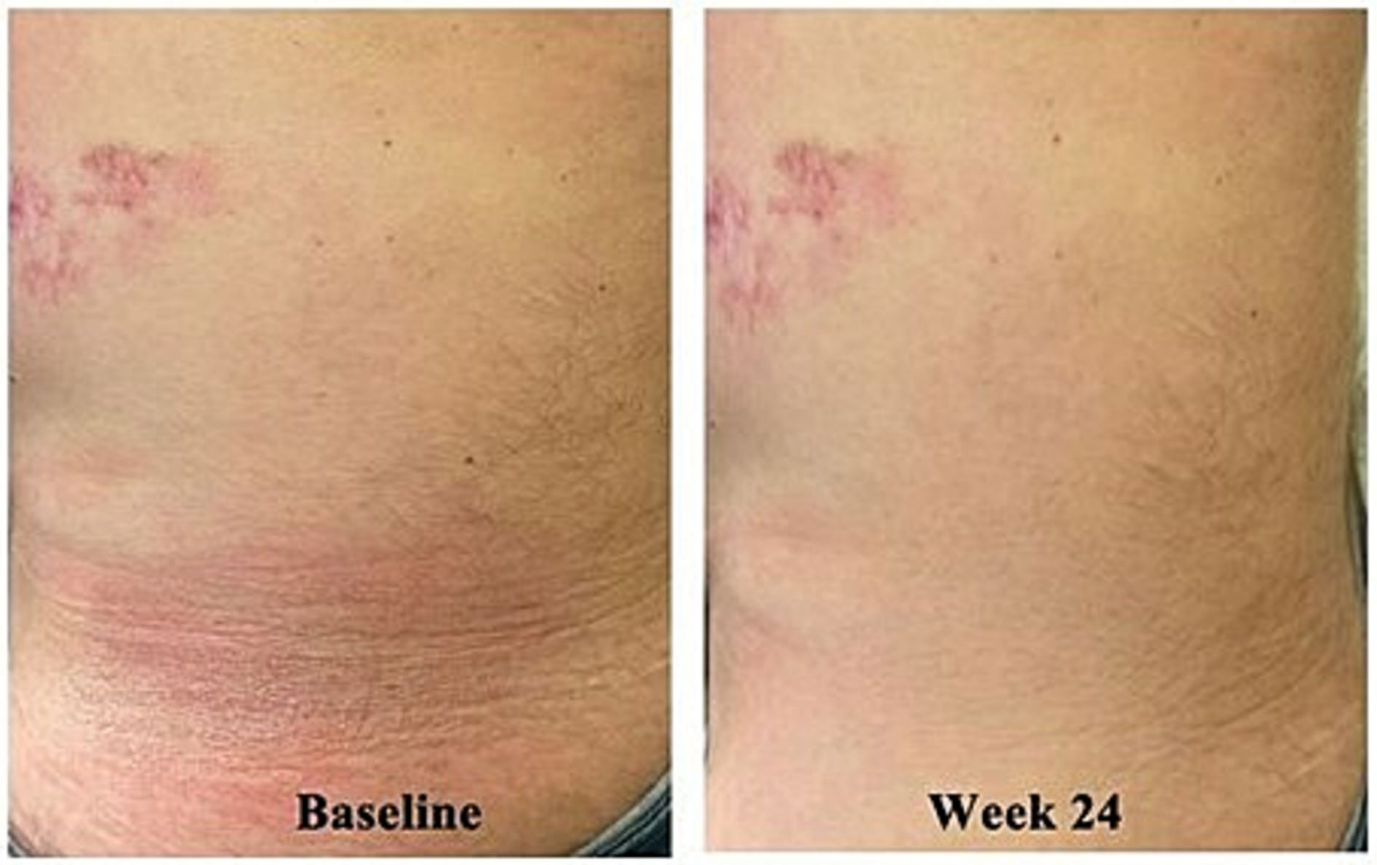
Case 5_48-year-old man. Forty eight-year-old kidney transplant recipient before and after 24 weeks of combination CL gel and TCS therapy (case courtesy of Mohannad Abu-Hilal).

The 48-year-old man with a past history of a kidney transplant presented with a 2-year history of persistent, non-itchy patches on his thighs, back, buttocks, and arms (BSA 25%). The patient was already taking systemic prednisone, tacrolimus, and mycophenolate mofetil (MMF) as a transplant recipient. At this time, a biopsy was done, and it revealed MF with typical epidermotropism (CD3+, CD4:CD8 ration of 4:1, with loss of CD7). Given his long-term use of systemic tacrolimus and MM, the decision was made to avoid phototherapy due to risk of UV exposure. Instead, the patient was started on topical TCS, clobetasol propionate daily and saw partial improvement with active lesions on lower back, thigh, and arms after 30 weeks of the TCS therapy. After discussing with the patient, transplant nephrologist and radiation oncologist, the patient was started on CL gel every other day in alternation with the clobetasol, in hopes to achieve full remission of his MF. On this regimen, the patient saw rapid remission of his patches within months (Figure 5).

This case highlights the targeted nature of CL gel SDT. It also provides an example of the utility of CL gel in complex patients on various systemic medications. As CL gel is not absorbed systemically, the risk of drug–drug interactions is negligible.

## Discussion

MF-CTCL is complex condition to diagnose and treat. Patients may go years prior to diagnosis and once diagnosed may cycle through many consecutive treatments until loss of response or tolerability (35). Durable remission is uncommon in MF-CTCL and treatment is often guided by symptom management and prevention of disease evolution. The goal of therapy is to provide itch relief, reduction of skin disease burden, cosmetic improvement, and possible reduction in rate of disease progression while maintaining or improving patient quality of life (1, 35). TCS are currently the most common first-line treatment for early-stage MF-CTCL (21, 22). However, TCS has significant side effects with long-term use and has few randomized, clinical trial data in MF use (16). Thus, there is an important unmet need for MF-CTCL treatment options in Canada (35). CL ointment has been available in Canada; however, both experts and clinicians across Canada have reported limited availability of the compounded ointment (35). Further, the ointment has been reported to be greasy and difficult for patients to apply. CL gel is a patient-friendly, accessible, safe, and effective SDT option for patients suffering from early-stage MF-CTCL.

Based on published data and clinical experience, CL gel should be made available as a first-line treatment for MF-CTCL in Canada. Both ESMO and EORTC have suggested CL gel in their first-line recommendations for MF-CTCL Stages IA-IIA (Table 3) (13, 22). In addition, CL gel has the highest level of evidence for its use (14, 21–23). The pivotal, study 201 was a large, randomized, controlled, multi-center trial conducted across 13 US academic centers that demonstrated that CL 0.02% topical gel (Ledaga™) was superior to CL 0.02% compounded ointment over a 12-month period in early-stage MF-CTCL patients (8). Study 202 was an open-label extension study of study 201 which investigated effects of 0.04% CL-gel in patients who did not achieve a complete response with the 0.02% concentration (36). The extension study demonstrated further clinical benefits with higher CL concentration and no unexpected toxicity or increase in skin-related adverse events (36). Further, real-world case series and expert experience suggest that CL-gel used 1–2 times weekly may also be efficacious as maintenance therapy for patients with full remission (27, 30).

CL-gel is a unique SDT as it has a precise, targeting effect on malignant T cells in the skin. *In vitro* studies have shown that healthy human T-cells were less susceptible to CL exposure than malignant T-cells (5). Chlormethine increases double-stranded DNA breaks in malignant T cells that lack the DNA repair mechanisms necessary to repair the breaks and continue DNA replication (5). Lack of these repair mechanisms in the malignant T-cells leads to apoptosis and cell death (5). This is supported by *in vitro* data showing that MF-CTCL stages IA-IIB skin T-cells have reduced expression of DNA-repair pathways with homologous recombination repair (HRR) genes being completely silenced (5). Thus, CL gel provides a precise therapeutic option that minimizes off-target effects that may lead to undesired adverse events. Bioanalytic testing results from pivotal trial samples show that CL gel does not penetrate past the epidermis. Thus, CL gel is not systemically absorbed. Lack of systemic absorption makes CL gel a potential adjuvant therapy for treating patches and plaques for advanced MF-CTCL as the gel will not produce any drug–drug interactions with other systemic therapies. CL gel is a targeted and versatile SDT that may be used as monotherapy in early-stage patients, adjuvant therapy in late-stage patients, and/or maintenance therapy for MF-CTCL patients in remission.

The CL gel’s most frequently observed adverse events are irritant and sometimes allergic dermatitis (8, 11). Experts agreed that the key to treatment success with CL gel was effectively management the skin irritation and allergy often associated with its side effect profile. In clinical trials, over 50% of patients experienced skin irritation with CL gel application (8). However, the cutaneous reaction does not negatively affect disease prognosis if appropriately managed. The MIDAS study provided evidence for combining TCS and CL-gel to effectively treat patients and prevent dermatitis reactions (12). While the MIDAS study did not show any impact on CL gel efficacy, some *in vitro* studies suggest that TCS may interfere with the inflammatory, anti-tumor immune response produced by chlormethine (24). Additional experience, research, and education on the CL gel will allow for development of personalized regimens for different types of patients, stages, and affected sites in MF-CTCL.

### Limitations

The expert opinions and real-world cases presented highlight the expert panel’s experience with CL gel in various patients under real-world conditions. All outcome measures were reported from providers in the clinic and reflect real-life data rather than data from controlled, clinical trial environment. Actual experience with CL gel may differ with each patient, provider, and treatment regimen. All expert opinions have been drawn from their own experiences with CL gel and do not encompass all possible reactions and outcomes of the gel’s use.

## Conclusion

Today, there are few treatment options for early-stage MF-CTCL in Canada. CL-gel is an accessible, safe, and reliable treatment for early-stage MF-CTCL. The novel gel formulation can be used as a first-line treatment in patients, such as monotherapy, or in combination with other SDT or systemic therapies. As an active ingredient, CL has years of data and experience in using compounded ointments or solutions. The CL-gel formulation provides easy application and improved efficacy and penetration in MF-affected skin. Pivotal trials demonstrate that the CL-gel has improved efficacy when compared to CL ointment. Further, patient and physician experiences confirm that the treatment’s gel formulation improves patient compliance and overall disease outcomes.
