# Supplementary material for: Case report: Canadian consensus on chlormethine gel use in mycosis fungoides-CTCL: literature review and real-world experience
Source: Front Med (Lausanne). 2024 Dec 16;11:1474030. doi: 10.3389/fmed.2024.1474030 (PMC11683787; doi:10.3389/fmed.2024.1474030)
Supplement: Supplementary file 1 [file Data_Sheet_1.docx]

Supplementary Materials

11 Draft Consensus Statements

1. Mycosis fungoides (MF) is the most common type of cutaneous T-cell lymphoma

(CTCL), representing almost half of all lymphomas arising in the skin.

2. In MF, skin infiltration of malignant T cells predominantly affects the epidermis.

3. Skin-related side effects of MF, such as dermatitis, can be managed through

appropriate strategies.

4. Currently, available therapies for early-stage MF are limited in Canada, without

topical agents previously indicated.

5. Chlormethine gel (CL gel) is a topical antineoplastic agent with phase II clinical trial

and real-world data demonstrating safety and efficacy as a treatment option for

adults with MF.

6. Post-hoc data analysis on CL gel indicated its clinical activity to be restricted to the

epidermis, making it particularly effective in early-stage disease.

7. CL gel is the skin-directed therapy with the highest level of recommendation for

early-stage (Stages IA, IB, and IIA) MF-CTCL in the 2018 European Society of Medical

Oncology guidelines.

8. A study indicated that the treatment with CL gel may result in higher and faster

response rates than treatment with CL ointment. The response rate continued to

improve with a longer duration of CL gel treatment.

9. CL gel can be applied for patients with stage IA and IB MF-CTCL as it provides an

easily administered, skin-directed treatment option.

10. Current clinical practice suggests CL gel is effective for treating early-stage MF-CTCL

and can be used as adjunctive therapy in advanced disease.

11. CL gel used for MF-CTCL has a predictable response and side effects with continued

efficacy after discontinuation. It provides a beneficial option for treating patients

with MF in Canada.1. Mycosis fungoides (MF) is the most common type of cutaneous T-cell lymphoma

(CTCL), representing almost half of all lymphomas arising in the skin.
